# Revolutionizing Early Detection: A Comprehensive Cognitive Screening Tool for ADRD Through Innovative Mobile App Technology

**DOI:** 10.1002/alz.087714

**Published:** 2025-01-09

**Authors:** Lenora W Smith, Charles O'Brien

**Affiliations:** ^1^ University of Alabama in Huntsville, Huntsville, AL USA

## Abstract

Currently, there are no curative treatments for Alzheimer’s Disease. It is crucial to develop screening tools that detect early signs of decline, allowing for timely interventions that may impede the progression of cognitive impairment. Neurospective is a mobile application platform designed to facilitate the early detection of cognitive decline, including mild cognitive impairment (MCI) and pre‐clinical Alzheimer’s disease and related dementias (ADRD). It utilizes a unique combination of engaging cognitive assessment games, diverse user data collection, and machine learning to establish a robust and accessible screening tool. Data Collection will include Cognitive Assessments (suite of mini‐games inspired by established screening tests like 3MS, MoCA, and SLUMS); User Metrics including app usage time, success rates, and reaction times, which provide additional insights into cognitive performance; and Surveys gathering demographic information, self‐reported cognitive impairment, and diagnosed ADRD. The primary aim of this study is to establish a relative measure of cognitive health within the population, eventually evolving into a reliable screening tool for early detection of cognitive decline. The innovation of this research is its accessibility, fostering widespread use, particularly in underrepresented populations facing limited access to traditional screening methods; its comprehensiveness with its diverse data collection, encompassing cognitive performance, usage metrics, and personal information, allowing a multifaceted approach to identifying early signs of cognitive decline; and evolving models including machine learning algorithms that continuously refine themselves based on user data, leading to increasingly accurate predictions of cognitive health and potential ADRD risk. Our target populations are people with pre‐clinical symptoms of ADRD and MCI. Our target data set is 25,000+ users with at least 10% survey completion. Both the app and the resulting dataset will be publicly available for further research and development. User privacy is protected through anonymity and voluntary participation. The Neurospective app presents a unique and promising approach to early detection of cognitive decline through its accessible mobile app format, comprehensive data collection, and evolving machine learning models. This project has the potential to significantly improve our understanding of cognitive health and contribute to earlier intervention for individuals at risk of ADRD.

